# Supplementary material for: Exploring the repository of de novo-designed bifunctional antimicrobial peptides through deep learning
Source: eLife. 2025 Mar 13;13:RP97330. doi: 10.7554/eLife.97330 (PMC11906162; doi:10.7554/eLife.97330)
Supplement: Supplementary file 1. — (a) Ablation study results of AMPredictor. Best metrics are marked in bold and second-best values are underlined. (b) Information about five used antiviral classifiers. (c) Sequences and novelty (BLAST E-value) of validated peptides. (d) The EC50 (μM) of AMPs inhibiting four enveloped viruses. (e) The selectivity index (SI) of three AMPs inhibiting four enveloped viruses. (f) Reported and predicted MICs of some recently mined AMPs. (g) Minimal inhibitory concentrations (μM) of P076-NH2 and P076. (h) The qRT-PCR primers. (i) System details of molecular dynamics simulations. (j) Binding free energy between peptides and the lipid bilayers. [file elife-97330-supp1.docx]

**Supplementary File 1**

**Exploring the repository of *de novo* designed bifunctional antimicrobial peptides through deep learning**

Ruihan Dong^1,2#^, Rongrong Liu^3#^, Ziyu Liu^3#^, Yangang Liu^4#^, Gaomei Zhao^5^, Honglei Li^6^, Shiyuan Hou^3^, Xiaohan Ma^3^, Huarui Kang^3^, Jing Liu^3^, Fei Guo^7^, Ping Zhao^4^, Junping Wang^5^, Cheng Wang^5,*^, Xingan Wu^3,*^, Sheng Ye^1,*^, Cheng Zhu^1,*^

^1^ Frontiers Science Center for Synthetic Biology (Ministry of Education), Tianjin Key Laboratory of Function and Application of Biological Macromolecular Structures, School of Life Sciences, Faculty of Medicine, Tianjin University, Tianjin 300072, China

^2^ Center for Quantitative Biology, Academy for Advanced Interdisciplinary Studies, Peking University, Beijing 100871, China

^3^ Department of Microbiology, School of Basic Medicine, Fourth Military Medical University, Xi’an 710000, Shaanxi, China

^4^ Department of Microbiology, Second Military Medical University, Shanghai 200433, China

^5^ State Key Laboratory of Trauma and Chemical Poisoning, Institute of Combined Injury of PLA, College of Preventive Medicine, Third Military Medical University (Army Medical University), Chongqing 400038, China

^6^ Tianjin Cancer Hospital Airport Hospital, Tianjin 300308, China

^7^ School of Computer Science and Engineering, Central South University, Changsha 410083, Hunan, China

^#^ These authors contributed equally.

^*^ Correspondence: wangctmmu@126.com (C.W.), wuxingan@fmmu.edu.cn (X.W.), sye@tju.edu.cn (S.Y.), cheng_zhu@tju.edu.cn (C.Z.)

**Supplementary Tables**

**Supplementary file 1a.** Ablation study results of AMPredictor. Best metrics are marked in bold and second-best values are underlined.

| **Fingerprint** | **Contact Map** | **ESM** | **RMSE** | **MSE** | **Pearson** | **CI** |
| --- | --- | --- | --- | --- | --- | --- |
| √ | × | × | 0.6431 | 0.4126 | 0.4965 | 0.6399 |
| × | √ | × | 0.7273 | 0.5290 | 0.1958 | 0.5406 |
| × | × | √ | 0.5386 | 0.2901 | 0.7032 | 0.6906 |
| √ | √ | × | 0.6543 | 0.4281 | 0.4768 | 0.6540 |
| √ | × | √ | 0.5356 | 0.2869 | 0.6961 | 0.6942 |
| × | √ | √ | 0.5636 | 0.3176 | 0.6494 | 0.7177 |
| **√** | **√** | **√** | **0.5348** | **0.2860** | **0.7072** | **0.7294** |

**Supplementary file 1b.** Information about five used antiviral classifiers.

| **No.** | **Name** | **Encoding** | **Model** |
| --- | --- | --- | --- |
| 1 | AVPpred | Motif, alignment, AAC, AAindex | SVM |
| 2 | DeepAVP | One-hot | CNN-LSTM |
| 3 | Deep-AVPpred | Pretrained language model | CNN |
| 4 | ENNAVIA | AAC, modlAMP, AAindex | MLP |
| 5 | AI4AVP | PC6 | CNN |

**Supplementary file 1c.** Sequences and novelty (BLAST E-value) of validated peptides.

| **Name** | **Sequence** | **Predicted MIC** | **Length** | **E-value ^a^** | **E-value ^b^** |
| --- | --- | --- | --- | --- | --- |
| P089 | GLKALMKILALFMAKKIT | 1.644 | 18 | No hits | 4.1 |
| P386 | PIGLKLRAAIAKGLLHALLKGARAD | 1.857 | 25 | 7.5 | 7 |
| P120 | RWKKILGKAGKLLALRSGKLIL | 1.161 | 22 | 2.6 | 2.4 |
| P135 | KWKQFLKKLAKLIATRIAIIIKRRLK | 1.078 | 26 | 0.85 | 5.3 |
| P247 | KKLGKPLFKGLKKILKLFVKM | 1.450 | 21 | 5.3 | No hits |
| P026 | GIHKKLGPIAKKLLKKIAKL | 3.200 | 20 | 2.7 | No hits |
| P039 | GWKPILKKAAKIGKRLTGWLFLNSRVN | 2.270 | 27 | 0.29 | 1.2 |
| P070 | LKWWKKFGRKLARKFRM | 2.410 | 17 | 6.6 | No hits |
| P091 | GRMIRKMFKKLWKILRD | 2.480 | 17 | 5.3 | 4.7 |
| P127 | QKPKKDCGGNLLGMIKKFLK | 3.112 | 20 | No hits | 2.7 |
| P122 | IKARLKLKQRIKLIKIGKTFRRRRTDNM | 2.663 | 28 | No hits | 6.9 |
| P352 | RKFKYKFSKLKRVIFMAK | 3.141 | 18 | No hits | 9.9 |
| P105 | FTLKKLLKKGKKTRKLLTPLIKDHSDAM | 1.855 | 28 | No hits | No hits |
| P316 | MFKKILKKATKVIAGLTGHLFWGTRL | 1.317 | 26 | 0.58 | No hits |
| P244 | GTCKGLLKKLLKGMAKFILK | 1.766 | 20 | 6.8 | No hits |
| P302 | GKWQGLLKMIAKILAK | 1.973 | 16 | 0.37 | 0.58 |
| P185 | FRGRIGKILLKMLAQLAK | 2.103 | 18 | 2.3 | 8.3 |
| P019 | PRLAKLLKGGKYILKKITM | 3.482 | 19 | No hits | 0.048 |
| P252 | GMGSFRKLIKKLAKW | 3.491 | 15 | 2.4 | 2.1 |
| P213 | HKCYKWFIKMLRKFQ | 2.982 | 15 | 7.2 | No hits |
| P036 | KMKTFKKKFLKQFAKRLAGRIYPRFK | 2.325 | 26 | No hits | 4.7 |
| P018 | NWKPKIYRTAKKILKIAGKTL | 2.695 | 21 | 2.5 | 2.1 |
| P168 | KKKKFKKFSKGFQKTLMGMLCMSAAKAW | 2.321 | 28 | No hits | 9.1 |
| P214 | KQKIFQKLAARGILLPLAPFIMERIKW | 2.802 | 27 | No hits | No hits |
| P089 | GLKALMKILALFMAKKIT | 1.644 | 18 | No hits | 4.1 |
| P386 | PIGLKLRAAIAKGLLHALLKGARAD | 1.857 | 25 | 7.5 | 7 |
| P120 | RWKKILGKAGKLLALRSGKLIL | 1.161 | 22 | 2.6 | 2.4 |
| P135 | KWKQFLKKLAKLIATRIAIIIKRRLK | 1.078 | 26 | 0.85 | 5.3 |

^a^: E-value was from alignment with the training set.

^b^: E-value was from alignment with DRAMP.

**Supplementary file 1d.** The EC_50_ (μM) of AMPs inhibiting four enveloped viruses.

| **AMP** | **Generator** | **CHIKV** | **HTNV** | **DENV-2** | **HSV-1** |
| --- | --- | --- | --- | --- | --- |
| P001 | v1 | 1.62 | 2.36 | 5.15 | 2.35 |
| P002 | v1 | 0.37 | 2.08 | 0.99 | 0.93 |
| P076 | v1 | 2.03 | 2.26 | 4.48 | 2.73 |
| P135 | v1 | 6.70 | 0.97 | 0.09 | 0.28 |
| P244 | v2 | 42.08 | 2.64 | 1.96 | 2.30 |

**Supplementary file 1e.** The selectivity index (SI) of three AMPs inhibiting four enveloped viruses.

| **AMP** | **CHIKV** | **HTNV** | **DENV-2** | **HSV-1** |
| --- | --- | --- | --- | --- |
| P001 | 37.27 | **50.36** | 11.71 | 25.72 |
| P002 | **149.21** | 31.75 | **51.05** | **59.01** |
| P076 | 22.64 | 15.68 | 14.90 | 16.90 |

**Supplementary file 1f.** Reported and predicted MICs of some recently mined AMPs.

| **No.** | **Peptide** | **Sequence** | **Ref.** | **Reported MIC** | **Predicted MIC** |
| --- | --- | --- | --- | --- | --- |
| 1 | BigDynorphin | YGGFLRRIRPKLKWDNQKRYGGFLRRQFKVVT | ^1^ | 14.58 | 7.89 |
| 2 | Apelin-1 | ERPVNLTMRRKLRKHNCLQRRCMPLHSRVPFP | ^1^ | 41.67 | 13.87 |
| 3 | Apelin-36 | LVQPRGSRNGPGPWQGGRRKFRRQRPRLSHKGPNPF | ^1^ | 29.17 | 7.75 |
| 4 | vWF-PQR19 | PQRMSRNFVRYVQGLKKKK | ^1^ | 63.50 | 29.52 |
| 5 | INTb-FTR26 | FTRGKLMSSLHLKRYYGRILHYLKAK | ^1^ | 25.00 | 6.28 |
| 6 | HSA-GKA38 | GKASSAKQRLKCASLQKFGERAFKAWAVARLSQRFPKA | ^1^ | 48.92 | 27.66 |
| 7 | CXC19-VKE44 | VKELIKKWEKQVSQKKKQKNGKKHQKKKVLKVRKSQRSRQKKTT | ^1^ | 76.00 | 0.76 |
| 8 | FIBg-TWK25 | TWKTRWYSMKKTTMKIIPFNRLTIG | ^1^ | 76.00 | 3.79 |
| 9 | SCUB1-MPF22 | MFPRSFIKLLRSKVSRFLRPYK | ^1^ | 60.37 | 4.43 |
| 10 | SCUB1-SKE25 | SKEMFPRSFIKLLRSKVSRFLRPYK | ^1^ | 22.92 | 4.61 |
| 11 | SCUB3-KHK26 | KHKEMLPKSFIKLLRSKVSSFLRPYK | ^1^ | 20.83 | 5.87 |
| 12 | SCUB3-MLP22 | MLPKSFIKLLRSKVSSFLRPYK | ^1^ | 18.23 | 3.36 |
| 13 | SFRP1-KKI32 | KKIVPKKKKPLKLGPIKKKDLKKLVLYLKNGA | ^1^ | 16.67 | 7.41 |
| 14 | NAPP-LIR38 | LIRIPLHRVQPGRRILNLLRGWREPAELPKLGAPSPGD | ^1^ | 55.17 | 6.14 |
| 15 | NAPP-LIR23 | LIRIPLHRVQPGRRILNLLRGWR | ^1^ | 87.42 | 20.82 |
| 16 | Bomidin | MGRFKRFRKKFKKLFKKLS | ^2^ | 4.00 | 5.43 |
| 17 | CBPZ-GSK24 | GSKPWWWSYFTSLSTHRPRWLLKY | ^3^ | 3.00 | 10.41 |
| 18 | XDH-AVA32 | AVAKLPAQKTEVFRGVLEQLRWFAGKQVKSVA | ^3^ | 32.00 | 23.51 |
| 19 | c_AMP660 | IFFRRNKKMAVKVAINGFGRIGRLAFRQMF | ^4^ | 10.00 | 14.56 |
| 20 | c_AMP575 | GRYIAKINPDNKKFKTMPSGKKRKGHKMATHKRKKRLRKNRHKKK | ^4^ | 2.00 | 1.49 |
| 21 | c_AMP1043 | KQKTLKKVWKLSEKVLIFASAFAKKAGAAEATLVL | ^4^ | 10.00 | 10.02 |
| 22 | c_AMP67 | AMTLRKRKFAWYVLSSSLKWLIKKAKKIGVQVCGFE | ^4^ | 20.00 | 4.80 |
| 23 | c_AMP69 | AMTSRKRKFVWYVLSSSLKWLIKKAKKIGVQVCGFE | ^4^ | 10.00 | 4.66 |
| 24 | c_AMP2041 | SVIWRKLFFIFIKRSGNWIKKVEKRQNLL | ^4^ | 20.00 | 48.78 |
| 25 | c_AMP250 | DRDRPECSTMVKYEQKLPSLGKYALKRAIKIKFGRK | ^4^ | 10.00 | 4.40 |
| 26 | c_AMP518 | GINLKRKGNIMKKVKNIFHKIANADPMIWGYVMLSESK | ^4^ | 25.00 | 6.29 |
| 27 | c_AMP593 | GVPMGSVIKKRRKRMAKKKHRKLLRKTRHQRRNKK | ^4^ | 25.00 | 2.16 |
| 28 | c_AMP1655 | RGTCYNRVGLIIRNFSKLKGKKV | ^4^ | 20.00 | 13.73 |

**Supplementary file 1g.** Minimal inhibitory concentrations (μM) of P076-NH_2_ and P076.

| **AMP** | ***S. aureus*** | | ***A. baumannii*** | |
| --- | --- | --- | --- | --- |
|  | ATCC 25923 | MRSA | ATCC 17978 | MDRAB |
| P076-NH_2_ | 53.12 | 106.32 | 53.12 | 53.12 |
| P076 | 6.64 | 13.29 | 0.21 | 0.21 |

**Supplementary file 1h.** The qRT-PCR primers.

| **Virus** | **Gene** | **Primers (5’-3’)** |
| --- | --- | --- |
| HTNV | S | Forward: GAGCCTGGAGACCATCTG  Reverse: CGGGACGACAAAGGATGT |
| CHIKV | E | Forward: TCTATAACATGGACTACCCGCCC  Reverse: AGCCAGATGGTGCCTGAGAGT |
| HSV-1 | VP16 | Forward: AATGTGGTTTAGCTCCCGCA  Reverse: CCAGTTGGCGTGTCTGTTTC |
| DENV-2 | NS5a | Forward: GGTTTTGGGAGCTGGTTGAC  Reverse: ACTCTAAGAAGCGTGCTCCA |

**Supplementary file 1i.** System details of molecular dynamics simulations.

| **No.** | **Peptide** | **Membrane** | **Box dimension (nm)** | **Atoms** |
| --- | --- | --- | --- | --- |
| 1 | P001 | Virus (POPC:POPE:POPS:POPI:DSM:cholesterol = 10:4:1:2:1:2) | 8.66 * 8.66 * 11.60 | 81,660 |
| 2 | P002 |  | 8.66 * 8.66 * 11.41 | 80,720 |
| 3 | P076 |  | 8.66 * 8.66 * 11.61 | 81,940 |
| 4 | P001 | Inner membranes of G- bacteria (POPE:POPG:TOCL1 = 7:2:1) | 8.13 * 8.13 * 11.60 | 73,644 |
| 5 | P002 |  | 8.13 * 8.13 * 11.41 | 72,702 |
| 6 | P076 |  | 8.13 * 8.13 * 11.61 | 73,721 |
| 7 | P001 | Outer membranes of G- bacteria (outer: 35 ECLIPA; inner: 75 PPPE, 20 PVPG, 5 PVCL2) | 8.05 * 8.05 * 12.54 | 84,979 |
| 8 | P002 |  | 8.15 * 8.15 * 11.88 | 82,483 |
| 9 | P076 |  | 8.07 * 8.07 * 12.46 | 84,922 |

**Supplementary file 1j.** Binding free energy between peptides and the lipid bilayers.

| **Peptide** | **Membrane** | $\boldsymbol{\Delta}$**G_polar_** | $\boldsymbol{\Delta}$**G_nonpolar_** | $\boldsymbol{\Delta}$**G_bind_** |
| --- | --- | --- | --- | --- |
| P001 | Virus | -193.00 ± 16.11 | -44.52 ± 2.99 | -229.65 ± 14.35 |
| P002 | Virus | -136.94 ± 22.66 | -53.60 ± 5.12 | -190.54 ± 18.02 |
| P076 | Virus | -195.71 ± 8.81 | -34.98 ± 8.73 | -230.69 ± 8.31 |
| P001 | G- inner membrane | -408.93 ± 35.39 | -47.97 ± 10.82 | -456.89 ± 29.24 |
| P002 | G- inner membrane | -289.58 ± 49.75 | -70.68 ± 31.58 | -360.26 ± 19.00 |
| P076 | G- inner membrane | -404.77 ± 2.89 | -61.02 ± 10.90 | -465.79 ± 11.39 |

**References**

1. M. D. T. Torres, M. C. R. Melo, L. Flowers, O. Crescenzi, E. Notomista and C. de la Fuente-Nunez, Mining for encrypted peptide antibiotics in the human proteome, *Nat Biomed Eng*, 2022, **6**, 67-75.

2. R. Liu, Z. Liu, H. Peng, Y. Lv, Y. Feng, J. Kang, N. Lu, R. Ma, S. Hou, W. Sun, Q. Ying, F. Wang, Q. Gao, P. Zhao, C. Zhu, Y. Wang and X. Wu, Bomidin: An Optimized Antimicrobial Peptide With Broad Antiviral Activity Against Enveloped Viruses, *Front Immunol*, 2022, **13**, 851642.

3. J. Maasch, M. D. T. Torres, M. C. R. Melo and C. de la Fuente-Nunez, Molecular de-extinction of ancient antimicrobial peptides enabled by machine learning, *Cell Host Microbe*, 2023, **31**, 1260-1274 e1266.

4. Y. Ma, Z. Guo, B. Xia, Y. Zhang, X. Liu, Y. Yu, N. Tang, X. Tong, M. Wang, X. Ye, J. Feng, Y. Chen and J. Wang, Identification of antimicrobial peptides from the human gut microbiome using deep learning, *Nat Biotechnol*, 2022, **40**, 921-931.
